# Publisher Correction: A network linking scene perception and spatial memory systems in posterior cerebral cortex

**DOI:** 10.1038/s41467-021-23781-x

**Published:** 2021-06-02

**Authors:** Adam Steel, Madeleine M. Billings, Edward H. Silson, Caroline E. Robertson

**Affiliations:** 1 Department of Psychology and Brain Sciences, Dartmouth College, Hanover, NH USA; 2 Psychology, School of Philosophy, Psychology, and Language Sciences, University of Edinburgh, Edinburgh, EH8 9JZ UK

**Keywords:** Cortex, Hippocampus, Visual system

Correction to: *Nature Communications* 10.1038/s41467-021-22848-z, published online 11 May 2021.

The original HTML version of this Article was updated shortly after publication because the previous HTML version linked to an incorrect Supplementary Information file.

## Supplementary information

Supplementary Information

