# Supplementary material for: A network linking scene perception and spatial memory systems in posterior cerebral cortex
Source: Nat Commun. 2021 May 11;12:2632. doi: 10.1038/s41467-021-22848-z (PMC8113503; doi:10.1038/s41467-021-22848-z)
Supplement: Supplementary file 1 — Supplementary Information [file 41467_2021_22848_MOESM1_ESM.pdf]

A network linking perception and memory systems in posterior cerebral cortex.

Supplementary Information

Adam Steel[1], Madeleine M. Billings[1], Edward H. Silson[2], Caroline E. Robertson[1]

[1]Department of Psychology and Brain Sciences, Dartmouth College, Hanover, NH, 03755

[2]Psychology, School of Philosophy, Psychology, and Language Sciences, University of Edinburgh, Edinburgh, UK EH8 9JZ

Corresponding author: Adam Steel, Department of Psychology and Brain Sciences, Dartmouth College, 3 Maynard Street, Hanover, NH, 03753; email: adam.steel@dartmouth.edu; tel: (202) 640 9340

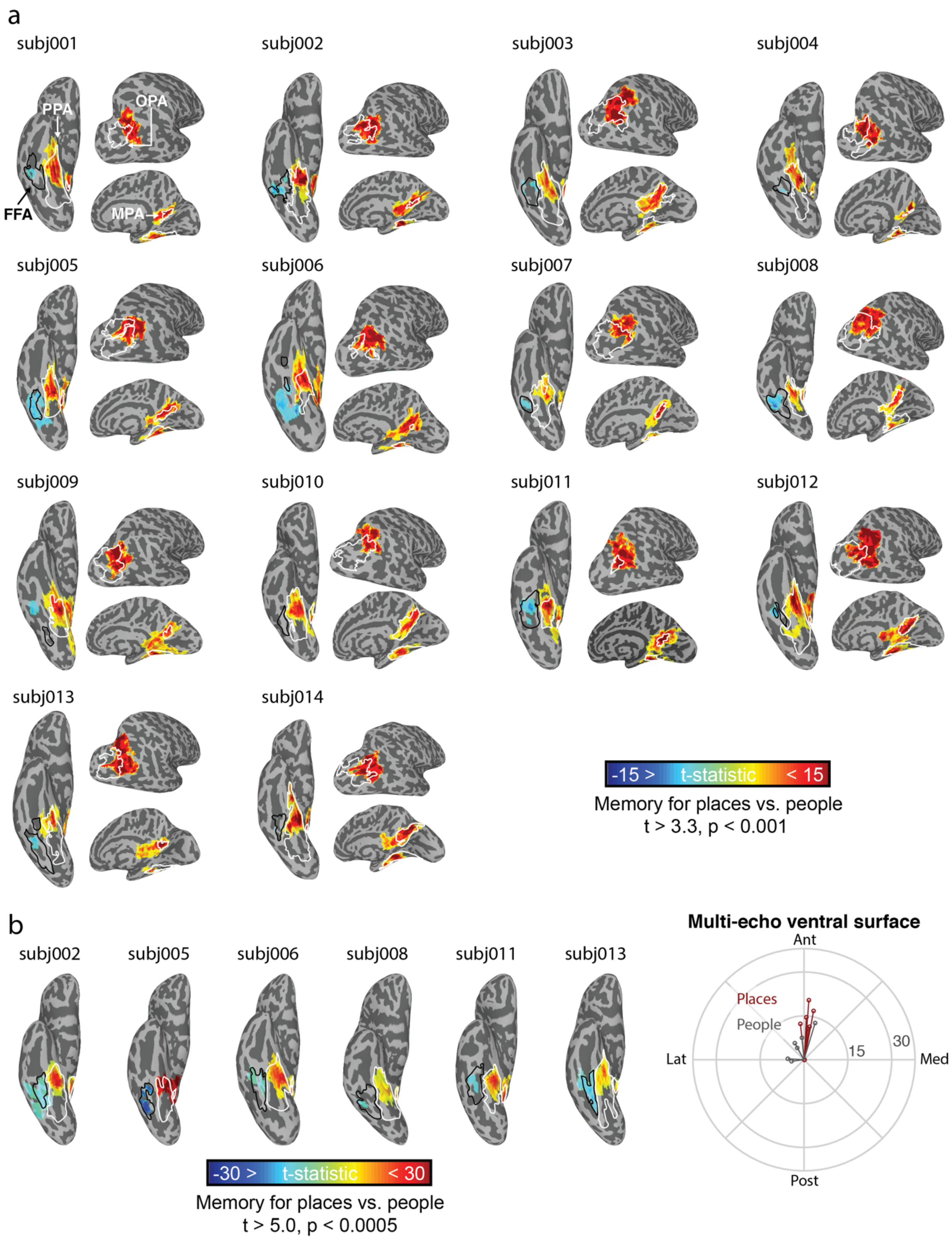
a
subj001
PPA
OPA
FFA
MPA
subj002
subj003
subj004
subj005
subj006
subj007
subj008
subj009
subj010
subj011
subj012
subj013
subj014
-15 >
t-statistic
< 15
Memory for places vs. people
t > 3.3, p < 0.001
b
subj002
subj005
subj006
subj008
subj011
subj013
-30 >
t-statistic
< 30
Memory for places vs. people
t > 5.0, p < 0.0005
Multi-echo ventral surface
Ant
Places
People
Lat
15
30
Med
Post

Supplementary Fig. 1. Place-memory selective activation is anterior to scene-selective perceptual activation in all participants. *a. In each participant, scene-selective perceptual areas (parahippocampal place area [PPA], occipital place area [OPA], and medial place area [MPA]) were localized by comparing BOLD activation when viewing images of places compared to people); the white outlined region shows each scene-selective area (PPA, OPA, MPA) thresholded at vertex-wise $p < 0.001$. Place-memory selective areas were localized by comparing BOLD activation when participants recalled personally familiar places versus personally familiar faces. These maps were thresholded at vertex-wise $p < 0.001$. In all participants and each cortical surface, the memory activation falls significantly anterior to scene perception activation. Face-selective perception (grey outline) and face-selective memory (cool colors) areas on the ventral surface are shown for comparison thresholded at (vertex-wise $p < 0.001$). At this threshold, people-memory activation on the ventral surface was only visible in 12/14 participants on the right hemisphere and 13/14 participants in the left hemisphere (not shown). In contrast to scene areas, the center of mass of people-memory was not anterior to face-perception. b. Signal dropout from the air-tissue interface of the ear canal is known to obscure activity in the lateral and anterior temporal lobe, and therefore could prevent us from observing an anterior bias during face memory recall activity relative to perception of faces. To ensure this was not the case, we replicated the comparison of perception and memory localizers using multi-echo fMRI in a subset of participants, with a short-TE (11 ms) to mitigate the dropout artifact* [108–110]*. We were able to replicate the anterior bias in place-memory selective activation (relative to activation during scene-perception) in the replication cohort. In addition, we did not observe any consistent anterior bias for people memory activation relative to activation during face perception. All participants unthresholded activation maps for both perception and memory localizers can be found in Supplementary file 1.*

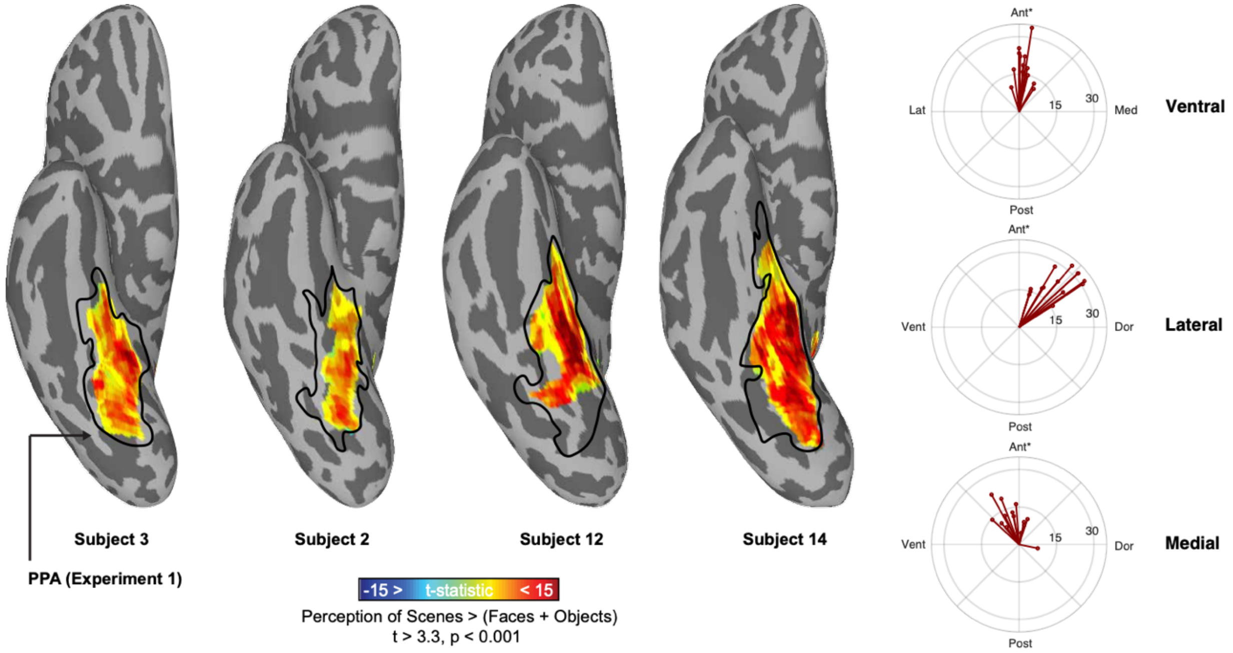

Supplementary Fig. 2. Contrasting scenes versus faces and objects does not impact the location of scene-perceptual areas. *The anterior bias of the place-memory areas relative to scene-perception areas persists when using a more conservative contrast to define scene areas (scenes > faces + objects)*[41]*, instead of the contrast used in Experiment 1 (scenes > faces). Left. Four example participants demonstrate the minimal impact of including visually objects when defining PPA. PPA defined by comparing blocks of scenes versus faces (as in Experiment 1) is outlined in black, and the PPA defined by comparing blocks scenes versus faces and objects is shown in hot colors. There was not significant shift in the weighted center-of-mass of PPA between the two ROI definitions (Left hemisphere: $t(13) = 0.99$, $p = 0.34$, Right hemisphere: $t(13) = 0.96$, $p = 0.35$), nor was there any difference in the center-of-mass of the two definitions of MPA (Left hemisphere: $t(13) = 0.37$, $p = 0.71$, Right hemisphere: $t(13) = 1.65$, $p = 0.12$). The weighted center of mass of OPA was shifted significantly posterior when objects were included in the contrast (Left hemisphere: $t(13) = 2.87$, $p = 0.013$, Right hemisphere: $t(13) = 2.22$, $p = 0.045$), and thus the original contrast would have been more difficult to observe an anterior shift of memory relative to perception. Right. Polar plots comparing scene-perception and place-memory areas. The center of the polar plot refers to the weighted center-of-mass of the scene-selective regions defined by contrasting scenes versus faces and objects in each participant, and each data point refers to the weighted center-of-mass of the place-memory area. The center-of-mass of the place-memory area was significantly anterior to the weighted center-of-mass of the scene-perception area on all surfaces (Medial surface -- Left hemisphere: $t(13) = 7.00$, $p < 0.0001$, Right hemisphere: $t(13) = 5.24$, $p = 0.00016$; Ventral surface -- Left hemisphere: $t(13) = 10.27$, $p < 0.0001$, Right hemisphere: $t(13) = 6.97$, $p < 0.0001$; Lateral surface -- Left hemisphere: $t(13) = 16.67$, $p < 0.0001$, Right hemisphere: $t(13) = 9.89$, $p < 0.0001$). The polar plots are ordered with ventral (top), lateral (middle), and medial (bottom). Anterior is towards the top of the figure for all surfaces, and the distance is given in millimeters.*

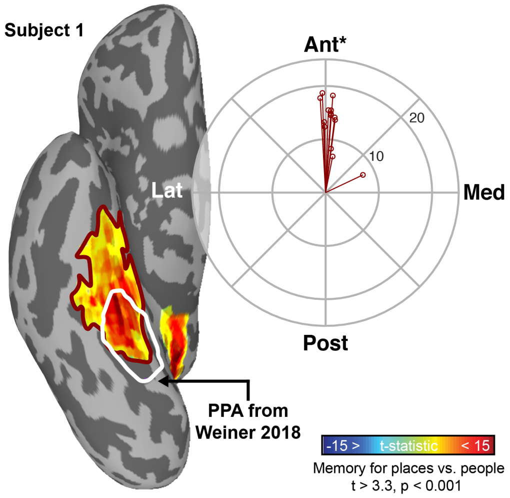

Supplementary Fig 3. The ventral place-memory area falls anterior to probabilistically-defined PPA. *Left. Data from an example participant. The ventral place-memory area (hot colors) is anterior to the most probable location of PPA[41]. Right. Polar plot showing the center of mass of the most probable location of PPA (Polar plot center) compared to the weighted center of mass of the ventral place memory area (data points). Consistent with the individually localized PPA (Figure 1), the center of mass of the ventral place memory area is anterior to the probabilistically defined PPA.*

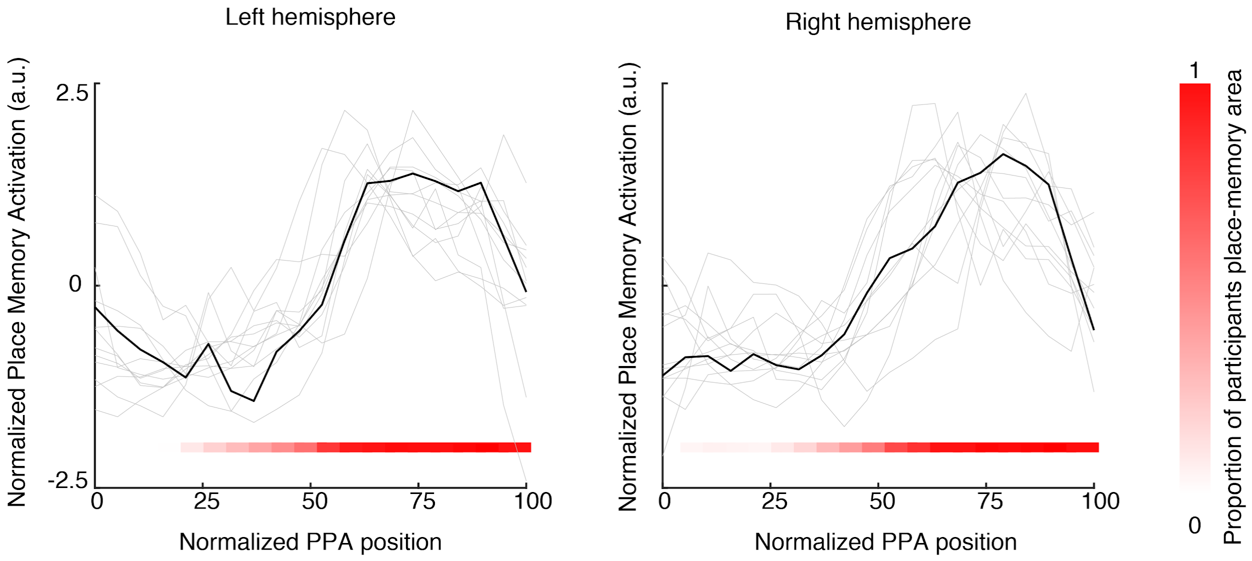

Supplementary Fig 4. Normalized place memory response along the posterior-anterior axis of the ventral scene-perception area (PPA). *In each participant (light grey lines), PPA was divided into 20 evenly spaced bins, and we calculated the mean place-memory activity (place-memory versus people-memory) from each bin. The place-memory activity from each participant was then demeaned and normalized, and the group average was calculated (black line).* ***Note that the y-axis shows demeaned and normalized place-memory responses, and therefore the direction of response (positive/negative) should not be interpreted.*** *At each bin, the red heatmap shows the proportion of participants with a place-memory area vertex within a given bin. At the group level, in both the left and right hemisphere, there was an apparent transition from perceptual to memory-related processing from approximately 50%-70% of the distance along the anterior-posterior axis of PPA. However, at the individual participant level, the transition was often sharper. It is impossible to estimate the true steepness of the transition between scene-perception and place-memory activity, however, due to the inherent smoothness of fMRI data. As such, these results should be interpreted with caution.*

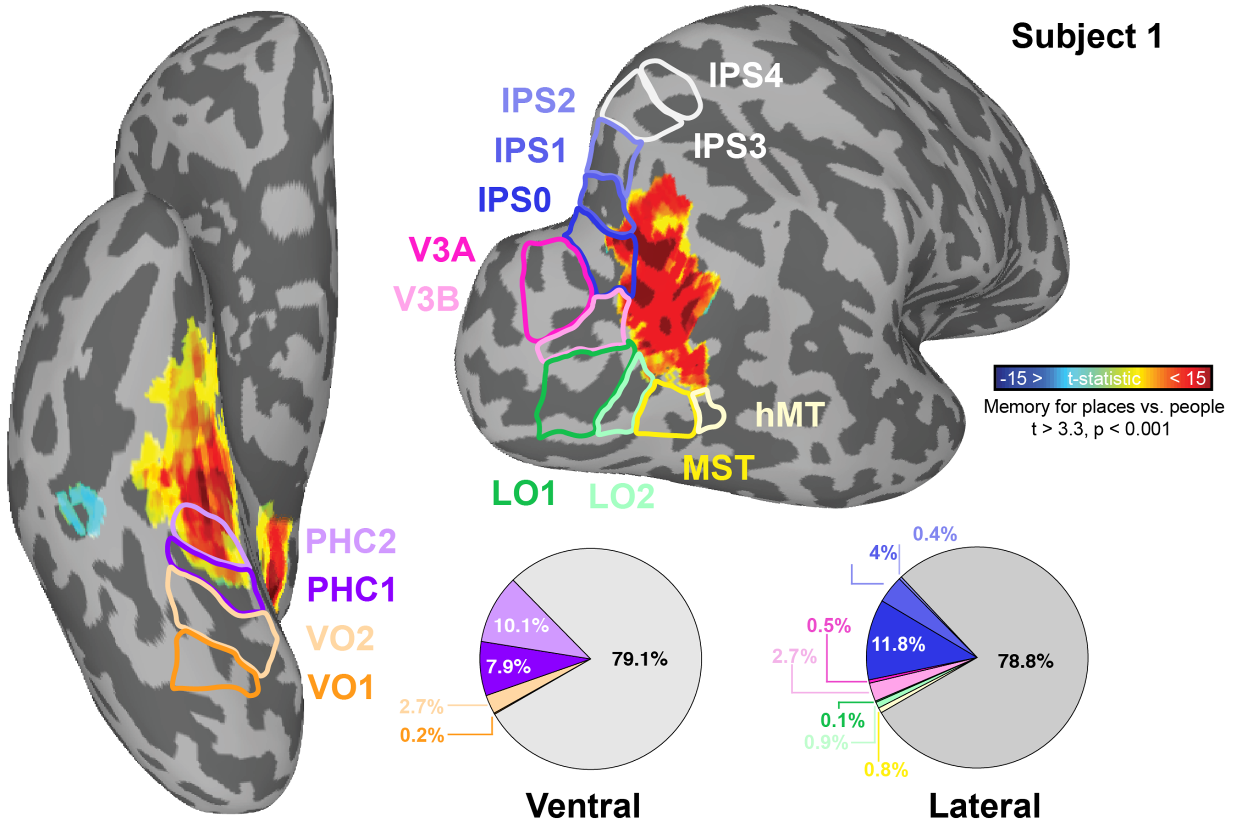

Supplementary Fig 5. Minimal overlap between cortical retinotopic maps and the place memory areas on the ventral and lateral surfaces. *The overlap between the maximum probability project of cortical retinotopic maps from Wang et al. 2014 overlaid on one individual participant's ventral and lateral place-memory activity. The place-memory areas on the ventral surface fall largely anterior to the retinotopic regions PHC1 and PHC2. On the lateral surface, the place-memory area primarily fell outside of retinotopic cortex. Inset. Group-average pie charts showing the proportion of surface vertices of the place memory areas contained within each retinotopic region at the individual participant-level. Across all participants, the majority of vertices fell outside of the cortical retinotopic maps. The medial surface is not shown due to the lack of retinotopic maps in medial parietal cortex.*

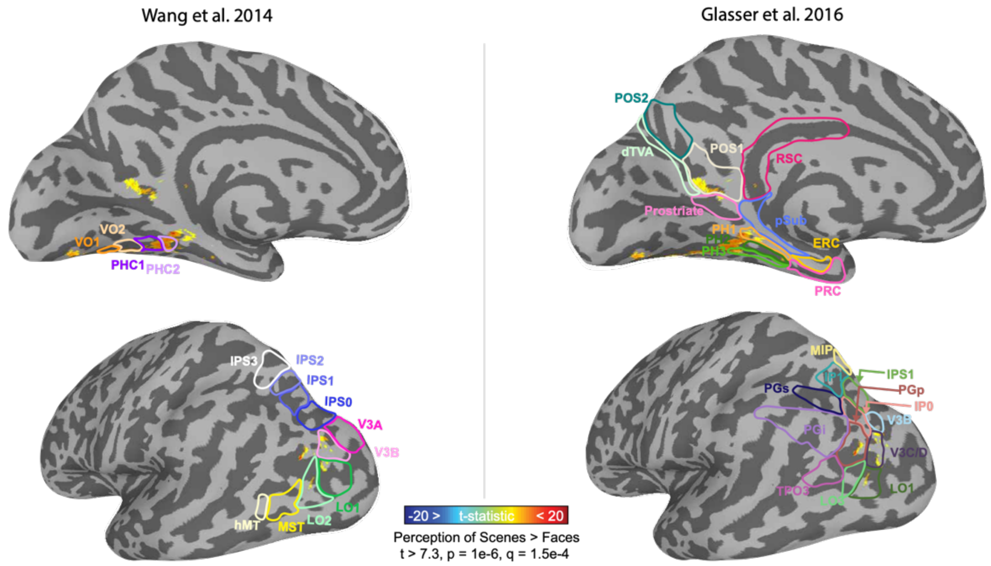

Supplementary fig. 6. Location of scene perception activity in relation to known functional and anatomical landmarks. left. Unlike the place-memory areas, the scene-perception fall largely within retinotopic cortex. We conducted a group analysis of the scene-perception localizer (place > people memory recall; thresholded at vertex-wise t > 7.3, p = 1e-6) and compared the resulting activation to the most probable location of the cortical retinotopic maps using the atlas (overlaid) from Wang et al. 2014. Right. Unlike the place-memory areas, the scene-perception areas fall in areas of cortex associated with perception based on their location relative to a widely used anatomical parcellation (Ventral: PHC 1-3, Medial: dorsal transitional visual area, Lateral: LO1/LO2, V3; Glasser et al. 2016).

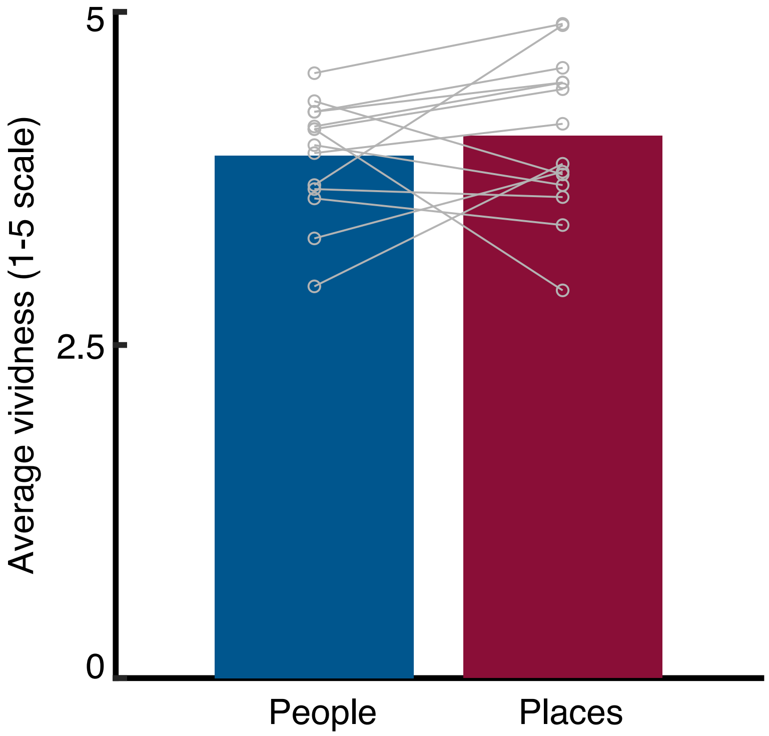

Supplementary Fig.7. Participants reported no difference in vividness between imagery for people and places. *Outside of the scanner, participants rated each personally familiar stimulus for vividness of visual imagery on a scale of 1-5, where 1 indicates no imagery possible and 5 indicates "as if you were seeing the <stimulus>." There was no significant difference in vividness between people and place stimuli (t(13) = 0.92, p = 0.373), suggesting that the topographical difference between place and people imagery activation was not due to differences of vividness between the stimulus categories. Additionally, we sought to rule out that the richness of recollection might impact the activity of the scene-perception and place-memory areas. So, we tested whether the "vividness" of a memory covaried with the magnitude of the place-memory network response on a given trial during Experiment 1 using a linear mixed effects model with Network (scene-perception/place-memory), stimulus type (person/place), hemisphere (left/right), and vividness as factors. We found no overall effect of vividness in any region (Main effect of Vividness -- Lateral: F(1,3716) = 0.7647, p = 0.3819; Ventral: F(1,3716) = 0.1657, p = 0.684; medial -- F(1,3716) = 0.0001, p = 0.99), nor did we find any interaction between vividness and stimulus type (Vividness x Stimulus type interaction -- lateral: F(1,3716) = 0.4437, p = 0.5054; ventral: F(1,3716) = 1.0179 p = 0.3131; medial: F(1,3716) = 1.5674, p = 0.2107) or vividness and network (Vividness x Network interaction -- lateral: F(1,3716) = 0.001, p = 0.97; ventral: F(1,3716) = 0.525, p = 0.46; medial: F(1,3716) = 0.016, p = 0.90).*

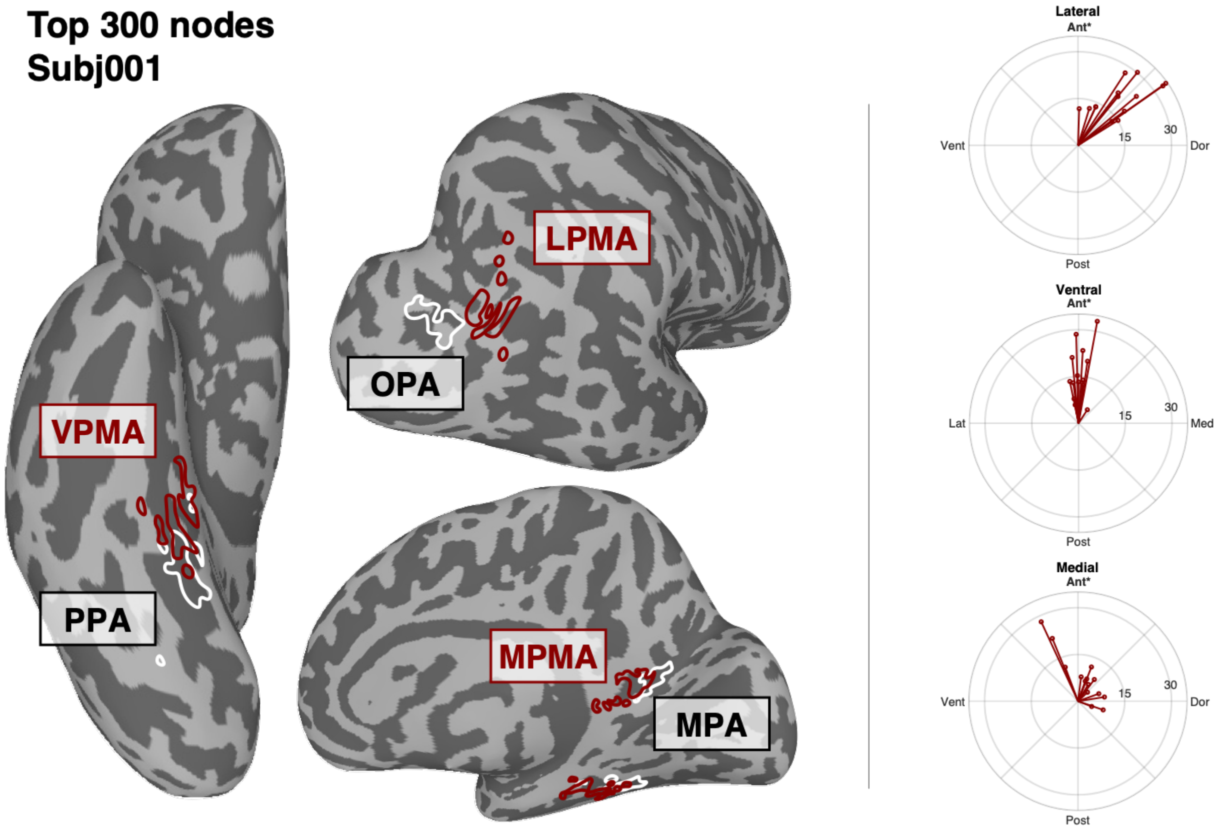

Supplementary Figure 7. **Anterior shift of place-memory relative to scene perception remains consistent after constraining to maximally selective surface vertices.** (Left). Top 300 in each participant most scene-selective surface vertices were used for as regions of interest for Experiments 2-4. (Right). Across the lateral, medial and ventral surfaces, we compared the weighted center-of-mass of both the scene-perception and place-memory selective areas after constraining the regions to the top 300 most scene-perception/place-memory preferring surface vertices. Using the constrained ROI definitions, we still observed a significant anterior shift from scene-perception to place-memory on all cortical surfaces in both the left and right hemisphere (Lateral - left hemisphere: $t(13) = 9.35$, $p < 0.0001$, right hemisphere: $t(13) = 8.62$, $p < 0.0001$; Ventral - left hemisphere: $t(13) = 4.84$, $p = 0.0003$, right hemisphere: $t(13) = 7.45$, $p < 0.0001$; Medial - left hemisphere: $t(13) = 2.86$, $p = 0.013$, right hemisphere: $t(13) = 2.36$, $p = 0.034$).

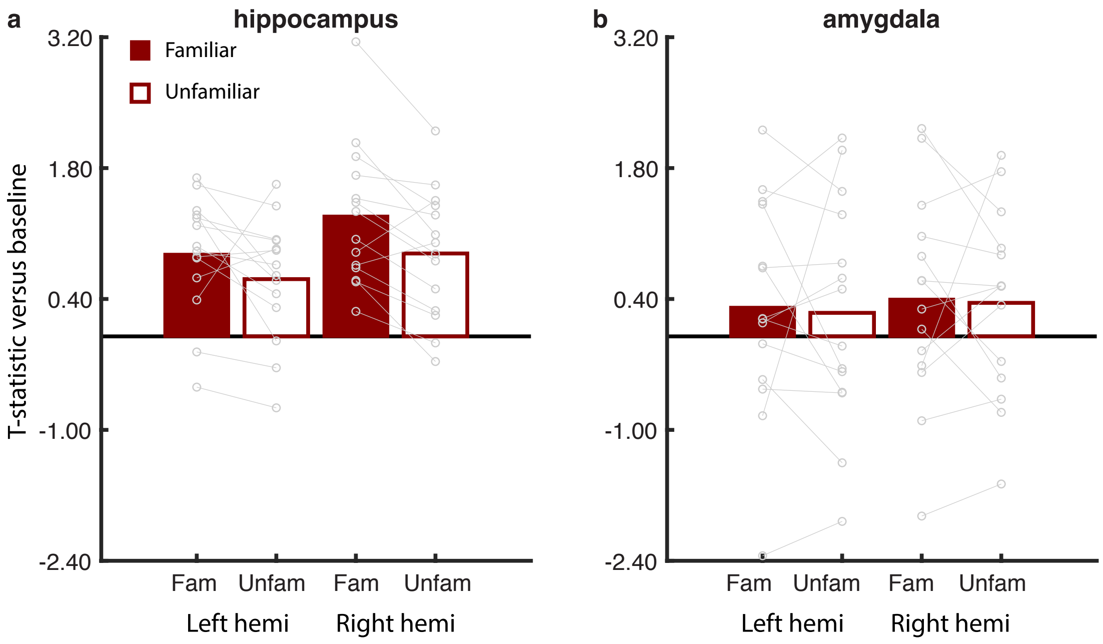

Supplementary Fig. 9. Hippocampus responds preferentially to familiar place movies, while amygdala does not. *As an exploratory analysis, we tested how the hippocampus, which we expected would preferentially respond to familiar images, responded in Experiment 2, where participants viewed familiar and unfamiliar panning movies. The average T-statistic from all voxels within the hippocampus and amygdala (defined by Freesurfer segmentation) was extracted for each participant. We then compared the activation for each region separately using a linear mixed effects model with Hemisphere (left/right) and Familiarity (familiar/unfamiliar) as factors. a. As predicted, the hippocampus preferentially activated to familiar compared to unfamiliar stimuli (Main effect of Familiarity: $F(1,39)=8.14$, $p = 0.0069$; $t(13)=2.54$, $p = 0.004$). Activation was stronger in the right hemisphere compared to the left hemisphere (Main effect of Hemisphere: $F(1,39)=8.61$, $p = 0.0056$; $t(13)=2.38$, $p=0.0055$); however, there was no interaction between Hemisphere and Familiarity (Hemisphere x Familiarity interaction: $F(1,39)=0.33$, $p = 0.56$). b. In contrast, in the amygdala, there was no effect of Familiarity ($F(1,39)=0.29$, $p = 0.59$), Hemisphere ($F(1,39)=0.07$, $p = 0.78$), or interaction between Hemisphere and Familiarity ($F(1,39)=0.003$, $p = 0.95$), arguing against a simple account of attention modulating the response to familiar compared to unfamiliar stimuli.*

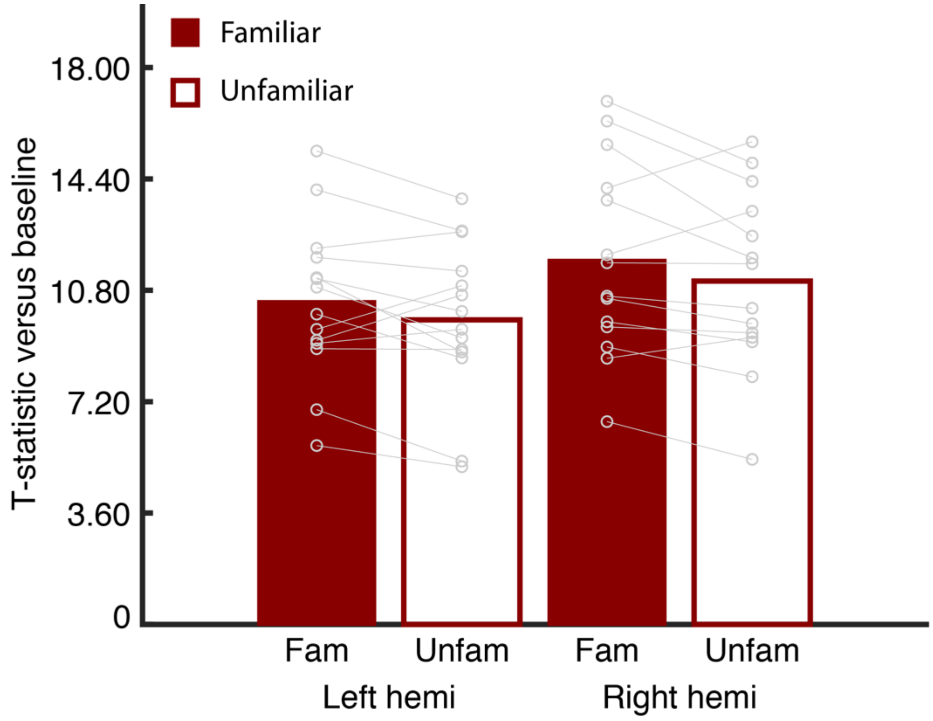

Supplementary Fig. 10. Early visual cortex responses to familiar and unfamiliar place movies do not differ. *One explanation for the increased activation to familiar compared to unfamiliar stimuli is that greater attention is being paid to familiar stimuli, causing a global increase in activation of visually-responsive areas. To confirm that this was not the case, we compared activation of the occipital pole (defined in each participant from their Freesurfer parcellation) when participants viewed familiar versus unfamiliar panning movies. We compared the responses using a mixed effects model with Hemisphere (lh/rh) and Familiarity (familiar/unfamiliar) as factors. We found that responses in the right hemisphere were stronger than the left hemisphere (Main effect of Hemisphere: $F(1,39) = 13.29$, $p = 0.0008$; $t39=3.65$, $p = 0.0008$). However, we found no effect of Familiarity ($F(1,39)=3.19$, $p = 0.08$) or interaction between Hemisphere and Familiarity ($F(1,39)=0.01$, $p = 0.91$), confirming that a simple attentional account cannot explain the familiarity effect observed in the place-memory areas.*

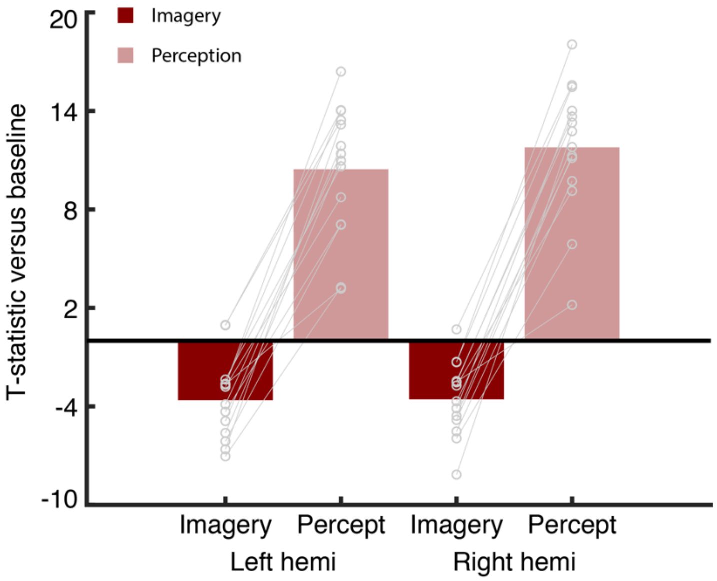

***Supplementary Fig. 11. Early visual cortex responds more strongly to perception than mental imagery**. As an exploratory analysis, we investigated the response in early visual cortex (occipital pole defined in individual participants from their Freesurfer segmentation) during perception of unfamiliar places and mental imagery of familiar places. We compared the responses using a mixed effects model with Hemisphere (lh/rh) and Task (imagery/perception) as factors. As expected, we found that early visual cortex responded significantly more to perception compared to mental imagery (Main effect of Task: $F(1,39) = 541.38$, $p < 0.0001$; $t(13) = 16.83$, $p < 0.0001$). There was no effect of Hemisphere ($F(1,39) = 1.22$, $p = 0.22$) or interaction between Hemisphere and Task ($F(1,39) = 1.05$, $p = 0.31$).*

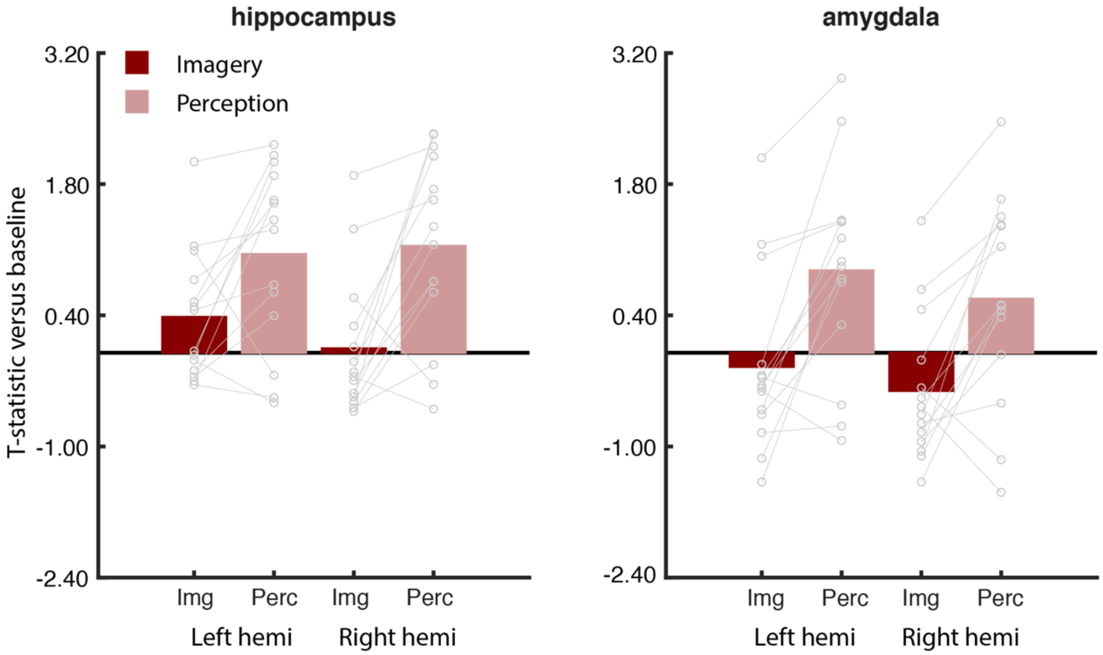

Supplementary Fig. 12. Both hippocampus and amygdala respond more strongly during perception compared to mental imagery. *As an exploratory analysis, we investigated whether the hippocampus and amygdala — subcortical areas implicated in memory processes — responded differently when viewing panning movies of unfamiliar places and mental imagery of familiar places. The average T-statistic from all voxels within the hippocampus and amygdala (defined by Freesurfer segmentation) was extracted for each participant. We then separately compared the activation of each region using a linear mixed effects model with Hemisphere (left/right) and Task (Imagery/Perception) as factors. Both the hippocampus and amygdala responded more strongly during perception compared to mental imagery (Hippocampus: $F(1,39)=28.67$, $p < 0.0001$ $t(13)=3.31$, $p < 0.0001$; Amygdala: $F(1,39)=37.22$, $p < 0.0001$; $t(13)=4.01$, $p < 0.0001$). Responses did not differ by hemisphere in either region (Hippocampus: $F(1,39)=0.57$, $p = 0.45$; Amygdala: $F(1,39)=2.86$, $p = 0.09$), and there was no interaction between Hemisphere and Task in either region (Hippocampus: $F(1,39)=1.65$, $p = 0.20$; Amygdala: $F(1,39)=0.02$, $p = 0.89$).*

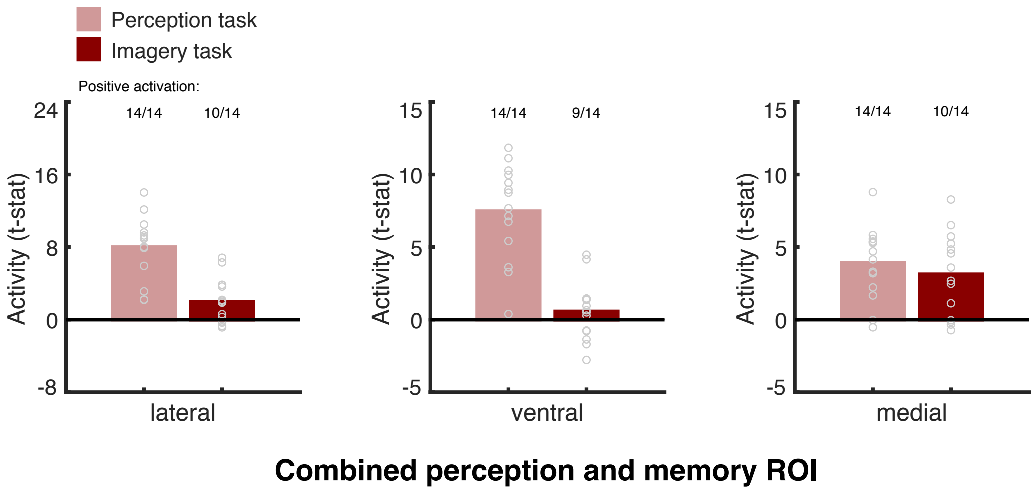

Supplementary Fig. 13. All cortical surfaces (lateral, ventral, medial) show positive activation during perception and imagery when scene-perception and place-memory areas are considered together. *Previous studies of mental imagery (e.g. [29]), have found that mental imagery and perception activate shared neural substrates. However, these studies have not considered the scene-perception and place-memory areas separately. Therefore, in order to relate our results to previous studies of mental imagery, the average t-statistic of the scene-perception and place-memory areas from each cortical surface were extracted and combined into a single "combined ROI". Consistent with prior work, we found that mental imagery positively activated the combined ROI in the majority of participants (lateral: 10/14, mean t-stat = 1.96; ventral: 9/14, mean t-stat = 0.56; medial: 10/14, mean t-stat = 3.12). Group activation of the lateral ($t(13) = 2.99$, $p = 0.011$) and medial ($t(13) = 4.08$, $p = 0.0013$) surfaces were significantly above zero during mental imagery. Despite the majority of participants showing positive activation, the ventral surface was not significantly above zero at the group level ($t(13) = 1.03$, $p = 0.32$).*

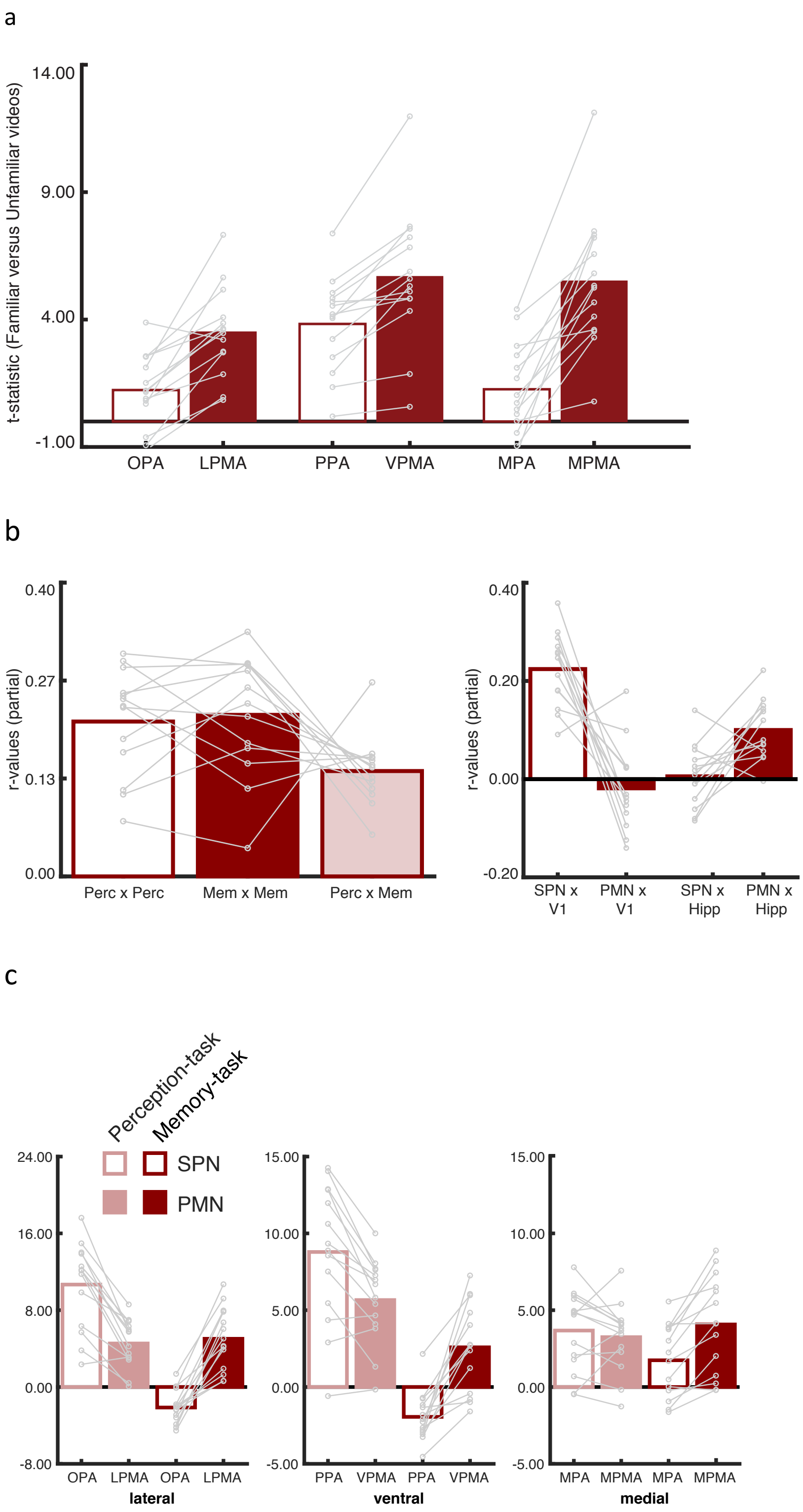

Supplementary Fig. 14. Results from all experiments remain unchanged when scene-perception areas are defined using the contrast scenes > faces + objects. *a. Experiment 2. Place memory areas exhibit an increased response to viewing panning movies of familiar places that is greater than the scene perception areas defined using the contrast Scenes > Faces + Objects. Lateral (LPMA v OPA): $t(13) = 2.65$, $p = 0.02$, $D = 0.7$; Medial (MPMA v MPA): $t(13) = 5.15$,*

$p = 0.00019$, $D = 1.13$; Ventral (VPMA v PPA): $t(13) = 3.18$, $p = 0.007$, $D = 0.85$). b. Experiment 3. Experiment 3. (left) The place memory areas and scene perception areas exhibit greater within network connectivity compared to between network connectivity when the scene perception areas defined using the contrast Scenes > Faces + Objects. P v PxM: $t(12) = 2.97$, $p = 0.01$, $D = 2.11$; M v PxM: $t(12) = 2.20$, $p = 0.048$, $D = 1.59$; P v M: $T(12) =3.4$, $p = 0.005$, $D = 0.93$. (right) The scene perception network is more connected to primary visual cortex than the place memory network, while the place memory network is more connected to hippocampus than the scene perception network when the scene perception areas are defined using the contrast Scenes > Faces + Objects. PxV1 - PxHipp v MxV1 - MxHipp: $t(12) = 6.3$, $p < 0.0001$, $D = 4.52$. c. Experiment 4. Place memory areas respond more when recalling familiar places compared to the scene perception areas when defined using the contrast Scenes > Faces + Objects. Lateral: Perception - $t(13) = 7.092$; $p < 0.0001$, $D = 2.3$, Memory - $t(13) = 8.509$; $p < 0.0001$, $D = 3.94$; Ventral: Perception - $t(13) = 4.803$; $p = 0.0003$, $D = 1.19$, Memory - $T(13) = 6.50$; $p < 0.0001$, $D = 2.77$; Medial: Perception - $t(13) = 0.83$; $p = 0.41$, $D = 0.25$, Memory - $T(13) = 4.82$; $p = 0.0003$, $D = 1.17$.

a

b

c

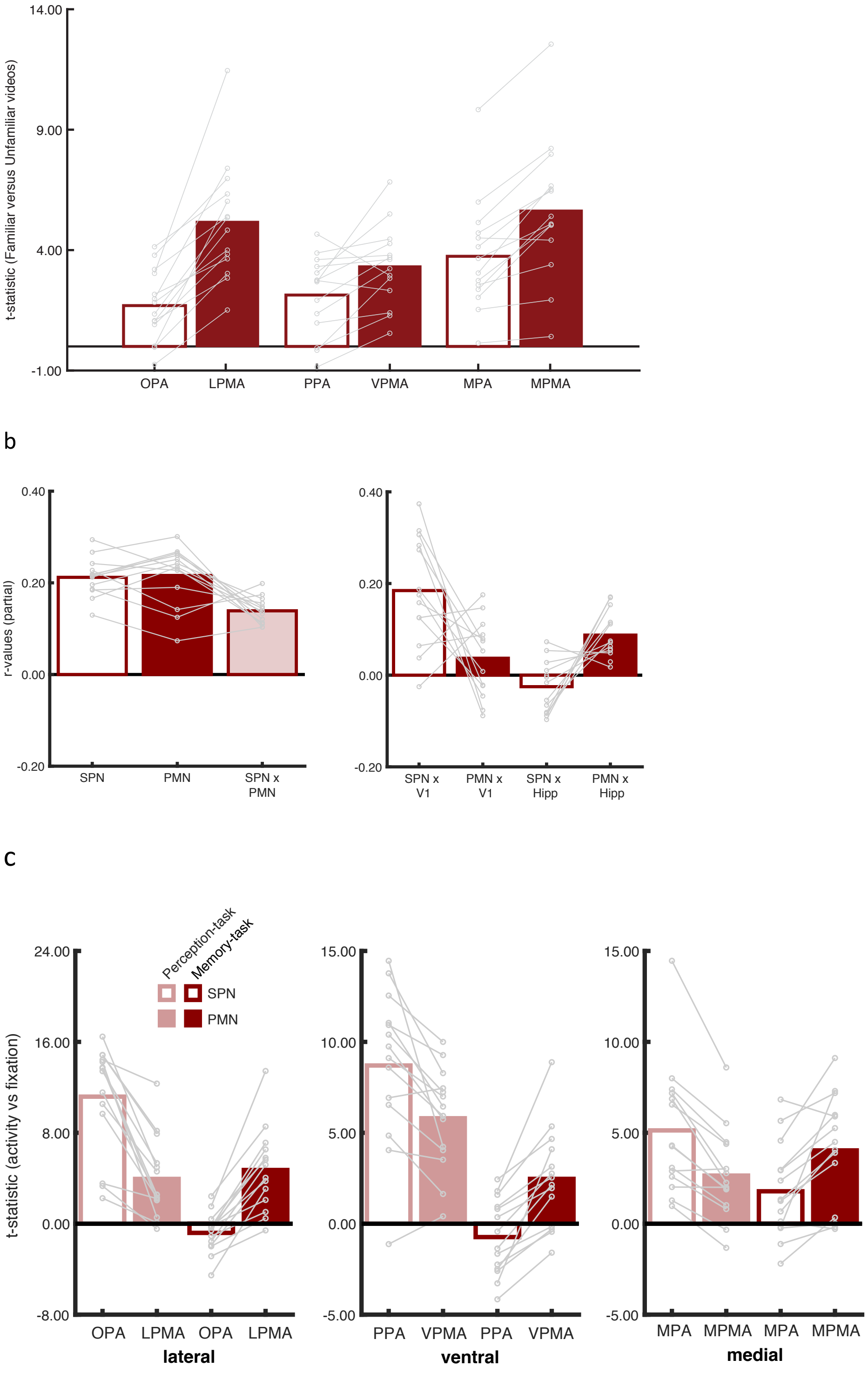

Supplementary Fig. 15. Results from all experiments remain unchanged when scene-perception areas are defined using the 100 vertices closest to the scene-perception and place-memory localizer activation peaks. *a. Experiment 2. Place memory areas exhibit an increased response to viewing panning movies of familiar places that is greater than the scene perception areas are defined using the 100 vertices closest to the localizer activation peak. Medial:*

t(13) = 5.98, p < 0.0001, D = 1.60; Ventral: t(13) = 4.80, p = 0.0003, D = 1.28; Lateral: t(13) = 4.32, p = 0.0008 , D = 1.28. b. Experiment 3. (Left) The place memory areas and scene perception areas exhibit greater within network connectivity compared to between network connectivity when the scene perception areas defined using the 100 vertices closest to the localizer activation peak. P v PxM: t(12) = 4.65, p = 0.0005, D = 2.84; M v PxM: t(12) = 3.45, p = 0.004, D = 2.14; P v M: t(12) =0.23, p = 0.79, D = 0.1. (Right) The scene perception network is more connected to primary visual cortex than the place memory network, while the place memory network is more connected to hippocampus than the scene perception network when the scene perception areas are defined using the contrast Scenes > Faces + Objects. PxV1 - PxHipp v MxV1 - MxHipp: t(12) = 5.8, p < 0.0001, D = 4.14. c. Experiment 4. Place memory areas respond more when recalling familiar places compared to the scene perception areas when the areas are defined using the 100 vertices closest to the localizer activation peak. Lateral: Perception – t(13) = 6.91, p < 0.0001 , D = 2.43, Memory – t(13) = 7.53, p < 0.0001, D = 2.7; Ventral: Perception – t(13) = 3.83, p = 0.002, D = 1.15, Memory – t(13) =6.34 , p < 0.0001, D = 1.85; Medial: Perception – t(13) =5.7 , p < 0.0001, D = 1.1; Memory – t(13) = 3.97, p = 0.0015, D = 1.12.
