# Supplementary material for: A network linking scene perception and spatial memory systems in posterior cerebral cortex
Source: Nat Commun. 2021 May 11;12:2632. doi: 10.1038/s41467-021-22848-z (PMC8113503; doi:10.1038/s41467-021-22848-z)
Supplement: Supplementary file 3 — Description of Additional Supplementary Files [file 41467_2021_22848_MOESM3_ESM.pdf]

Description of Additional Supplementary Files

File Name: Supplementary Movie 1
Description: All participants unthresholded activation maps. This file is a gif image displaying, alternatingly, the unthresholded activation maps for the scene perception (perception of scene versus faces) and place memory (memory recall of places versus people) localizer tasks. The anterior shift of memory relative to perception can be seen in all participants. Note that, for clarity, only the right hemisphere is shown because no significant interaction with hemisphere was found.

File Name: Supplementary Movie 2
Description: Example panning video of a familiar outdoor place used in Experiments 2 and 4 (Oxford, United Kingdom).

File Name: Supplementary Movie 3
Description: Example panning video of a familiar indoor place used in Experiments 2 and 4 (Hanover, NH, United States).

File Name: Supplementary Movie 4
Description: Example panning video of a familiar city street used in Experiments 2 and 4 (Yongkang, Zhejiang, China).

File Name: Supplementary Movie 5
Description: Example panning video of a familiar place used in Experiments 2 and 4 (Zandvoort, Netherlands).
