# Supplementary figures and images for: A network linking scene perception and spatial memory systems in posterior cerebral cortex
Source: Nat Commun. 2021 May 11;12:2632. doi: 10.1038/s41467-021-22848-z (PMC8113503; doi:10.1038/s41467-021-22848-z)

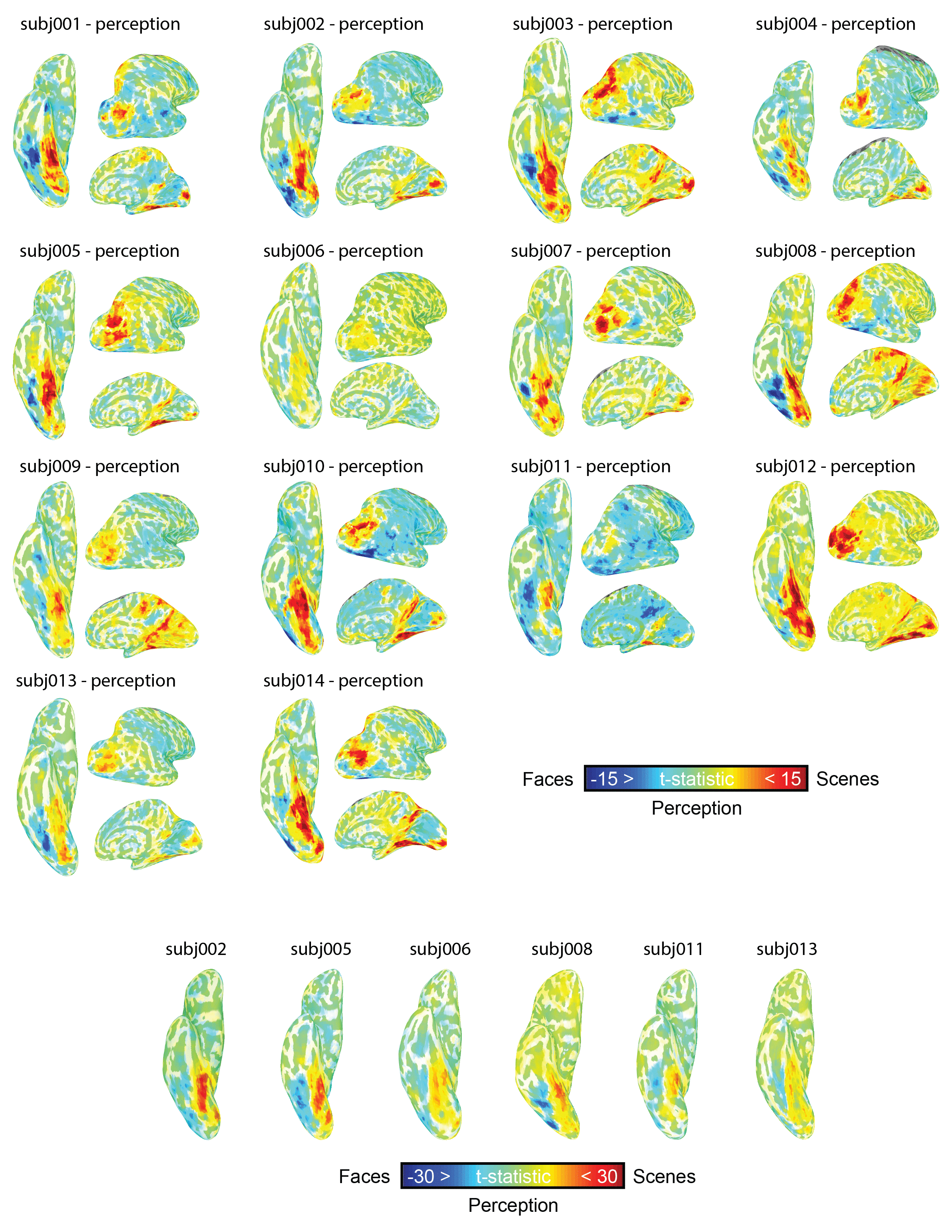

Supplement: Supplementary file 4 — Supplementary Videos 1–5 [file 41467_2021_22848_MOESM4_ESM.zip › ncomms_supplementary_videos (1)/Supplementary Movie 1.gif]
