# Supplementary material for: A network linking scene perception and spatial memory systems in posterior cerebral cortex
Source: Nat Commun. 2021 May 11;12:2632. doi: 10.1038/s41467-021-22848-z (PMC8113503; doi:10.1038/s41467-021-22848-z)
Supplement: Supplementary file 5 — Reporting Summary [file 41467_2021_22848_MOESM5_ESM.pdf]

nature research

Corresponding author(s): Adam Steel

Last updated by author(s): Mar 26, 2021

Reporting Summary

Nature Research wishes to improve the reproducibility of the work that we publish. This form provides structure for consistency and transparency in reporting. For further information on Nature Research policies, see our Editorial Policies and the Editorial Policy Checklist.

Statistics

For all statistical analyses, confirm that the following items are present in the figure legend, table legend, main text, or Methods section.

n/a	Confirmed	
☐	☒	The exact sample size (n) for each experimental group/condition, given as a discrete number and unit of measurement
☐	☒	A statement on whether measurements were taken from distinct samples or whether the same sample was measured repeatedly
☐	☒	The statistical test(s) used AND whether they are one- or two-sided<br>*Only common tests should be described solely by name; describe more complex techniques in the Methods section.*
☐	☒	A description of all covariates tested
☐	☒	A description of any assumptions or corrections, such as tests of normality and adjustment for multiple comparisons
☐	☒	A full description of the statistical parameters including central tendency (e.g. means) or other basic estimates (e.g. regression coefficient) AND variation (e.g. standard deviation) or associated estimates of uncertainty (e.g. confidence intervals)
☐	☒	For null hypothesis testing, the test statistic (e.g. F, t, r) with confidence intervals, effect sizes, degrees of freedom and P value noted<br>*Give P values as exact values whenever suitable.*
☒	☐	For Bayesian analysis, information on the choice of priors and Markov chain Monte Carlo settings
☒	☐	For hierarchical and complex designs, identification of the appropriate level for tests and full reporting of outcomes
☐	☒	Estimates of effect sizes (e.g. Cohen's d, Pearson's r), indicating how they were calculated

Our web collection on statistics for biologists contains articles on many of the points above.

Software and code

Policy information about availability of computer code

Data collection	Stimulus presentation: Psychopy3 (version 3.2.3)
Data analysis	FMRI data analysis: dcm2niix (v1.0.20190902), freesurfer (v6.0), AFNI (v20.3.02 'Vespasian'), FSL (6.0.1), custom MATLAB code.

For manuscripts utilizing custom algorithms or software that are central to the research but not yet described in published literature, software must be made available to editors and reviewers. We strongly encourage code deposition in a community repository (e.g. GitHub). See the Nature Research guidelines for submitting code & software for further information.

Data

Policy information about availability of data

All manuscripts must include a data availability statement. This statement should provide the following information, where applicable:

- Accession codes, unique identifiers, or web links for publicly available datasets
- A list of figures that have associated raw data
- A description of any restrictions on data availability

Data will be made available upon request. No databases were used for this study.

Field-specific reporting

Life sciences study design

All studies must disclose on these points even when the disclosure is negative.

Sample size	Sample size for Experiment 1 was determined by pilot testing of an independent group of participants approximately 4 weeks after Experiment 1 concluded. No power analysis was calculated prior to running the study. Post-hoc power analyses confirmed that the statistical tests were well-powered (Cohen's D > 0.92). These subjects then underwent Experiments 2-4.<br><br>The effect of interest in experiment 1 replicated successfully. This effect was observed in all participants.
Data exclusions	No data was excluded from any analysis
Replication	We independently replicate our findings in Experiment 1 using an advanced multiecho fMRI acquisition in a subset of participants. The statistical power of the effects observed in all other experiments is reported for reproducibility purposes. The power (Cohen's D) in all tests was greater than 0.92, suggesting that these effects are robust
Randomization	All experimental conditions were evaluated within-participant. Within participant, trial conditions were pseudorandomized to optimize contrast between conditions.
Blinding	Participants were unaware of the manipulation (single blind). The study design (within subject) made blinding not necessary.

Reporting for specific materials, systems and methods

We require information from authors about some types of materials, experimental systems and methods used in many studies. Here, indicate whether each material, system or method listed is relevant to your study. If you are not sure if a list item applies to your research, read the appropriate section before selecting a response.

Materials & experimental systems

n/a	Involved in the study	
☒	☐	Antibodies
☒	☐	Eukaryotic cell lines
☒	☐	Palaeontology and archaeology
☒	☐	Animals and other organisms
☐	☒	Human research participants
☒	☐	Clinical data
☒	☐	Dual use research of concern

Methods

n/a	Involved in the study	
☒	☐	ChIP-seq
☒	☐	Flow cytometry
☐	☒	MRI-based neuroimaging

Human research participants

Policy information about studies involving human research participants

Population characteristics	Fourteen adults (9 females; age=25.7±3.6 STD years old) participated in Experiments 1, 2, 4. Thirteen of these participants participated in Experiment 3 (females, age=25.9±3.6 STD years old). In addition, a subset of the original participants (N=6; 2 females, age=26.3±4.4 STD years old) completed the multi-echo replication of Experiment 1. Participants had normal or correct-to-normal vision, were not colorblind, and were free from neurological or psychiatric conditions. We confirmed that participants were able to perform mental imagery by assessing performance Vividness of Visual Imagery Questionaire. All participants score above 9 with eyes open, indicating satisfactory performance (mean=15.2, range=9.75-19.25).
Recruitment	Participants were recruited from Dartmouth Department of Psychology and Brain Sciences recruiting list-serve. We are not aware of any bias (self selection or other) that might impact the results.
Ethics oversight	Written consent was obtained from all participants in accordance with the Declaration of Helsinki and with protocol approved by the Dartmouth College Institutional Review Board (Protocol #31288).

Note that full information on the approval of the study protocol must also be provided in the manuscript.

Magnetic resonance imaging

Experimental design

Design type	Task fMRI: Event-related design, block-design. Task fMRI: Naturalistic movie watching.
Design specifications	Experiment 1) Scene perception localizer: 2 runs, 5 blocks/condition per run, 24 s blocks, no ISI. Place memory localizer: 4 runs, 9 trials/condition per run, 10 s trials, 4-8 s ISI.<br>Experiment 2: 6 runs, 8 trials/condition per run, 10 s trials, 4-8 s ISI.<br>Experiment 3: 11-minute continuous movie stimulus.

	Experiment 4: 6 runs, 8 trials/condition per run, 10 s trials, 4-8 s ISI.
Behavioral performance measures	No behavioral performance was collected during scanning.

Acquisition

Imaging type(s)	Functional, structural MRI
Field strength	3T
Sequence & imaging parameters	T1-weighted: magnetization-prepared rapid acquisition gradient echo (MPRAGE) imaging sequence was acquired (TR=2300 ms, TE=2.32 ms, inversion time=933 ms, Flip angle=8°, FOV=256 x 256 mm, slices=255, voxel size=1 x 1 x 1 mm).<br><br>In Experiments 1-4, single-echo T2*-weighted echo-planar images acquired using the following parameters: TR=2000 ms, TE=32 ms, GRAPPA=2, Flip angle=75°, FOV=240 x 240 mm, Matrix size=80 x 80, slices=34, voxel size=3 x 3 x 3 mm.<br><br>Multi-echo fMRI: The sequence parameters were: TR=2000 ms, TEs=[11.00, 25.33, 39.66, 53.99 ms], GRAPPA=3, Flip angle=75, FOV=240 x 240 mm, Matrix size=80 x 80, slices=40, Multi-band factor=2, voxel size=3 x 3 x 3 mm.
Area of acquisition	Single-echo EPI covering the temporal, parietal, and frontal cortices were acquired for experiments 1-4. Multiecho fMRI covered whole brain.

Diffusion MRI ☐ Used ☒ Not used

Preprocessing

Preprocessing software	AFNI version 19.3.16 (compiled Dec. 12, 2019)
Normalization	Data were not normalized, all analysis was conducted using regions of interest in subject native space
Normalization template	Group analyses were conducted on the suma standard mesh (std.141).
Noise and artifact removal	Experiments 1, 2, 4: Only motion parameters and baseline polynomials (3rd order) were used.<br><br>Experiment 2: ICA-based denoising was conducted by manually classifying noise (Graffanti et al. 2014).
Volume censoring	No censoring was performed

Statistical modeling & inference

Model type and settings	Experiments 1, 2, 4) First level: Mass univariate (AFNI 3dDeconvolve) to acquire beta-weights for each condition.<br><br>All second level analyses were conducted using ROI-based statistics
Effect(s) tested	Experiment 1) Block-wise beta values for scene versus face perception; Beta-values for place versus people memory recall trials.<br>Experiment 2) Beta-values for familiar versus unfamiliar place perception trials.<br>Experiment 4) Beta-values for mental imagery versus perception trials.

Specify type of analysis: ☐ Whole brain ☒ ROI-based ☐ Both

Anatomical location(s)	Scene-perception and place-memory ROIs were functionally defined for Experiments 1-4. Early visual cortex was defined using occipital pole ROI from Freesurfer parcellation. Hippocampus was defined for each participant using Freesurfer segmentation.
Statistic type for inference (See Eklund et al. 2016)	Whole-brain tests were FDR corrected to a conservative threshold (q = 0.00015).
Correction	Bonferonni FWE corrections were applied where appropriate.

Models & analysis

n/a	Involved in the study
☐	☒ Functional and/or effective connectivity
☒	☐ Graph analysis
☒	☐ Multivariate modeling or predictive analysis

Functional and/or effective connectivity	Experiment 2: Partial correlation was used.
